# Forebrain Deletion of αGDI in Adult Mice Worsens the Pre-Synaptic Deficit at Cortico-Lateral Amygdala Synaptic Connections

**DOI:** 10.1371/journal.pone.0029763

**Published:** 2012-01-23

**Authors:** Veronica Bianchi, Frédéric Gambino, Luca Muzio, Daniela Toniolo, Yann Humeau, Patrizia D'Adamo

**Affiliations:** 1 Dulbecco Telethon Institute at Division of Neuroscience, San Raffaele Scientific Institute, Milan, Italy; 2 Centre National de la Recherche Scientifique UPR3212, CNRS, University of Strasbourg, Strasbourg, France; 3 Département des Neurosciences Fondamentales, CMU, Genève, Suisse; 4 Division of Neuroscience, San Raffaele Scientific Institute, Milan, Italy; 5 Division of Genetics and Cell Biology, San Raffaele Scientific Institute, Milan, Italy and Institute of Molecular Genetics-CNR, Pavia, Italy; 6 Institut Interdiciplinaire de Neuroscience Centre National de la Recherche Scientifique UMR5297, University of Bordeaux, Bordeaux, France; Radboud University, The Netherlands

## Abstract

The *GDI1* gene encodes αGDI, which retrieves inactive GDP-bound RAB from membranes to form a cytosolic pool awaiting vesicular release. Mutations in *GDI1* are responsible for X-linked Intellectual Disability. Characterization of the *Gdi1*-null mice has revealed alterations in the total number and distribution of hippocampal and cortical synaptic vesicles, hippocampal short-term synaptic plasticity and specific short-term memory deficits in adult mice, which are possibly caused by alterations of different synaptic vesicle recycling pathways controlled by several RAB GTPases. However, interpretation of these studies is complicated by the complete ablation of *Gdi1* in all cells in the brain throughout development. In this study, we generated conditionally gene-targeted mice in which the knockout of *Gdi1* is restricted to the forebrain, hippocampus, cortex and amygdala and occurs only during postnatal development. Adult mutant mice reproduce the short-term memory deficit previously reported in *Gdi1*-null mice. Surprisingly, the delayed ablation of *Gdi1* worsens the pre-synaptic phenotype at cortico-amygdala synaptic connections compared to *Gdi1*-null mice. These results suggest a pivotal role of αGDI via specific RAB GTPases acting specifically in forebrain regions at the pre-synaptic sites involved in memory formation.

## Introduction

Human intellectual disability (ID), also referred to as Mental Retardation (MR), is a common human neurodevelopment disorder with onset early in postnatal life. This condition affects approximately 2–3% of the human population [1]. ID is classified based on intelligence quotient (IQ); the presence of other clinical features distinguishes syndromic ID (S-ID) from non-syndromic ID (NS-ID). Over the past 15 years, mutations in ∼ 40 genes have been associated with NS-ID and ∼ 80% of these are found on the X-chromosome. The identification and functional characterization of these genes have greatly enlarged our understanding of human cognition and intellect. Because NS-ID is characterized clinically only by intellectual impairment, the genes underlying this clinical condition are likely to be involved in learning and memory formation. Additionally, the functional study of these genes might help to understand neural development and provide treatment strategies for NS-ID.

The *GDI1* gene encodes αGDI, a protein that controls the cycling of RAB GTPase [2]. RAB GTPases are proteins involved in the control of intracellular traffic; they act as molecular switches between active GTP-bound and inactive GDP-bound conformations. The role of αGDI is to bind the RAB in its GDP-bound state from membranes and to maintain a cytosolic pool of GDP-associated RAB. The discovery of mutations in the *GDI1* [3] and *RAB39B* [4] genes in ID patients supports the importance of the intracellular trafficking mediated by the αGDI-RAB GTPase pathway in the development of cognitive function.

We have previously demonstrated that the lack of αGDI in *Gdi1*-null mice impaired hippocampal dependent forms of short-term memory, namely, the working and associative fear-related memory formation, and greatly reduced male aggression, thereby modifying social interaction [5].

We also showed that the absence of αGDI in the brain leads to alterations in the total number of hippocampal and cortical synaptic vesicles (SV) during synaptic differentiation at postnatal days (PND) 10 and 30. In adult mice at PND 90, the distribution of SV was altered at differentiated neuronal terminals, and the reserve pool (RP) appeared to be specifically affected [6]; this likely resulted in slow recovery after SV depletion and a short-term memory deficit in *Gdi1*-null mice.

Thus, the complete ablation of *Gdi1* leads to a complex phenotype that is most likely the result of alterations in the temporal and spatial functioning of αGDI and some of the associated RABs. Our previous results suggest that these alterations might be due to the reduced availability of the endocytic RABs RAB4 and RAB5, which are involved in slow SV recycling and in alterations of RAB3A, which is the main RAB protein mediating SV exocytosis. Nevertheless, other RAB proteins may be involved in the *Gdi1*-null phenotype, particularly proteins that have recently been shown to be associated with SV membranes [7].

A major limitation for the study of αGDI function in the central nervous system (CNS) and the interpretation of all results obtained from the analysis of *Gdi1*-null mice is the fact that the complete ablation of *Gdi1* occurs in all brain regions and all brain cell types throughout development in these mice. Moreover, the previously observed phenotype of the *Gdi1*-null mice may be due to alteration of several RAB GTPases and subsequent compensation.

To solve this problem, we used the CRE/lox *P* recombination system to restrict *Gdi1* knockout both spatially and temporally to postmitotic neurons of the anterior forebrain. These conditional *Gdi1* knockout mice were viable and developed without any obvious morphological defects. We show that the conditional *Gdi1* mice phenocopy the cognitive deficit and showed greatly reduced male aggression, as was previously observed in the *Gdi1*-null mice, confirming the pivotal role of αGDI in hippocampal, cortical and amygdala synapses in the process of memory formation. Surprisingly, the electrophysiological comparison at cortico-amygdala synaptic connections between the *Gdi1*-null and conditional *Gdi1* mice reveals a stronger pre-synaptic phenotype in conditional *Gdi1* mice. Because significant levels of recombination do not start until the end of the third postnatal week, when many of the CNS neuronal pathways have already been established, these results suggest that the regulation of RABs by αGDI directly modulates complex behavior that is important for cognitive function.

## Results

### Inactivation of αGDI occurs in specific brain regions during postnatal life

**Figure 1:**
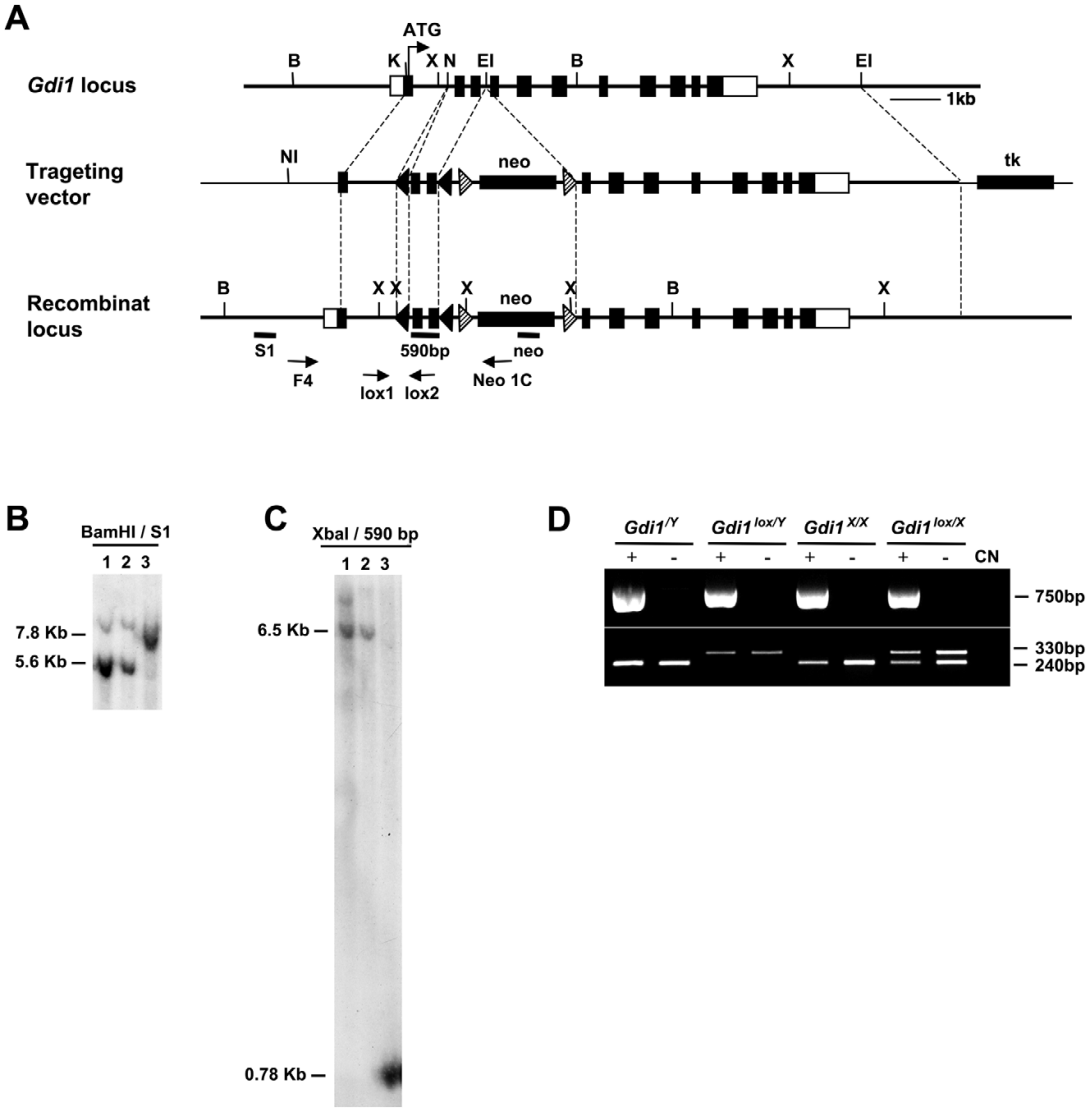
Gene targeting and generation of *Gdi1^flox/Y^* mice. (**A**) Scheme of the structural organization of the *Gdi1* gene (top), of the targeting vector (middle) and of the recombinant locus (bottom). Black boxes are coding exons or the indicated insertion cassettes; white boxes are 5′ and 3′ UTR regions. Restriction enzymes are BamHI (B), XbaI (X), NotI (NI), EcoRI (EI), NdeI (N) and KpnI (K). The position of the PCR primers for the screening of the G-418 resistant embryonic stems cell clones (F4; Neo1C) and for mice screening (Lox1; Lox2) are indicated by arrows. S1, neo and 590 bp are the probes for Southern blot analysis. (**B–C**) Southern blot analysis of the embryonic stem cell clone found positive by PCR (lane 3), compared with negative clones (lane 1–2). (**B**) Genomic DNAs were digested with BamHI, fractionated on agarose gel and hybridized with the probe 5′ S1 probe. The 5.6 Kb BamHI fragment corresponds to the wild type locus, the 7.8 Kb fragment to the recombinant locus, with neo insertion. (**C**) Genomic DNAs were digested with XbaI, fractionated on agarose gel and hybridized with the 590 bp probe, corresponding to the fragment cloned between lox *P* sites. The 6.5 kb XbaI fragment corresponds to the wild type locus and the 0.780 Kb fragment to the recombinant locus with the insertion of lox *P* sites. (**D**) PCR analysis of DNA extracted from the tails of mice, using the primers Lox1/Lox2 (330 bp correspond to the lox allele and 240 bp to the WT) and Cre1/Cre2 (750 bp correspond to the presence of the *CaMKII-Cre*-159 transgene).

To generate a conditional *Gdi1* mouse model, we generated a floxed allele of *Gdi1* by flanking exons 2 and 3 of the mouse *Gdi1* gene with two lox *P* sites using homologous recombination in ES cells (Fig. 1A). One homologous recombinant clone with the highest percentage of normal karyotypes (90%) was analyzed by Southern blot (Fig. 1B and C) and was injected into blastocysts. Derived germline targeted females offspring carrying one floxed allele (*Gdi1^lox/X^*) were first crossed with transgenic male mice expressing the tgFlp recombinase to excise the neomycin cassette and subsequently backcrossed into a C57BL/6N genetic background. All subsequent work was performed using *neo^−^* mice.

To inactivate *Gdi1* in the postnatal forebrain, we crossed heterozygous *Gdi1^lox/X^* female mice with the transgenic line *CaMKII-Cre*-159 [8] (provided from Dr. R. Brambilla, HSR, Milan-Italy) to generate *CaMKII-Cre^+^-Gdi1^flox/Y^* and *CaMKII-Cre^−^-Gdi1^lox/Y^* male mice (referred to as *Gdi1^flox/Y^* for *Gdi1* knockout and *Gdi1^lox/Y^* for wild-type mice) for all subsequent work.

**Figure 2:**
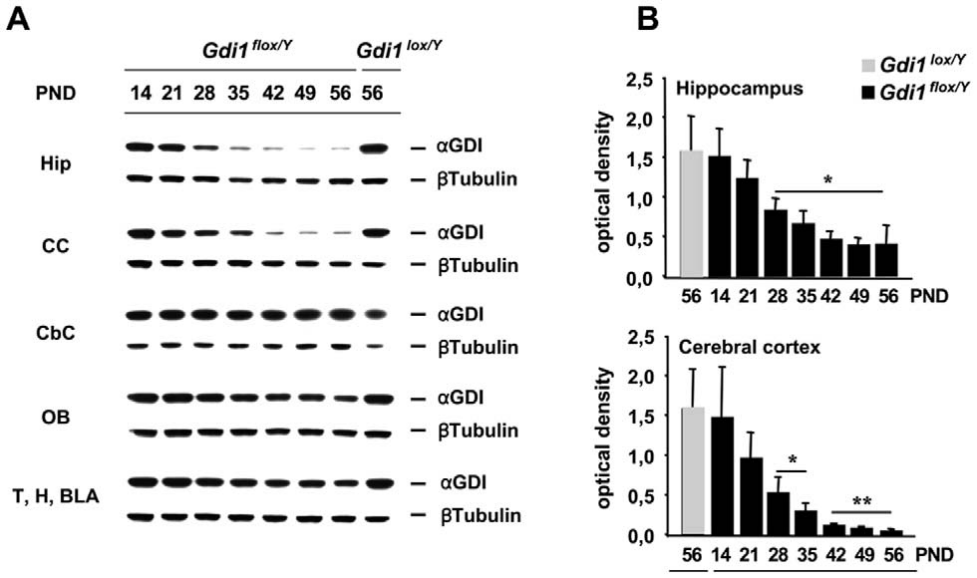
Brain region- and age-specific down regulation of αGDI on *Gdi1^flox/Y^* mice. Two to eight week old male mice were analysed. (**A**) Protein lysates were prepared from the indicated tissues (Hip: hippocampus, CC: cerebral cortex, CbC: cerebellum, OB: olfactory bulb, T: thalamus, H: hypothalamus and BLA: amygdala) at the indicated age (PND: post natal days), fractionated on 10% SDS–PAGE gels and analyzed with a commercial anti-GDI antibody. β-tubulin was used as loading control. (**B**) Quantitative analysis of residual αGDI normalized by β-tubulin in the hippocampus and cerebral cortex. Protein levels were quantified by measuring the intensity of the western blot signal with the Image Quant system. Values are expressed as mean ± SD from three independent animals at each age. Grey squares are *Gdi1^lox/Y^* animals and black squares are *Gdi1^flox/Y^*. *p<0.05, **p<0.01.

Western blot analysis was performed on several brain regions at different developmental stages to assess the regional and temporal pattern of αGDI inactivation driven by *CaMKII-Cre*-159 transgene activity *in vivo* (Fig. 2). At several postnatal days (ranging from PND 14 to 56), hippocampus (Hip), cerebral cortex (CC), olfactory bulbs (OB), cerebellum (CbC) and a mixture of the thalamus (T), hypothalamus (H) and amygdala (BLA) were dissected from *Gdi1^flox/Y^* and compared with *Gdi1^lox/Y^* littermate mice (PND 56) (Fig. 2). No difference in the αGDI protein level was observed in the OB (mean ± SD; *Gdi1^flox/Y^* 1.44±0.37; *Gdi1^lox/Y^* 1.5±0.38), CbC (mean ± SD; *Gdi1^flox/Y^* 4.14±0.97; *Gdi1^lox/Y^* 4.48±1) and T-H-BLA (mean ± SD; *Gdi1^flox/Y^* 1.2±0.33; *Gdi1^lox/Y^* 1.51±0.05) homogenates, confirming that recombination was not active in these brain regions, as previously reported [8]. At PND 28, αGDI was still detectable but down regulated by 50% in the Hip (t-test, p = 0.05) and by 65% in CC (t-test, p = 0.02). The down-regulation reached 80% at PND 56 in the Hip (t-test, p = 0.01) and 98% in CC (t-test, p = 0.005), as shown in Fig. 2B.

**Figure 3:**
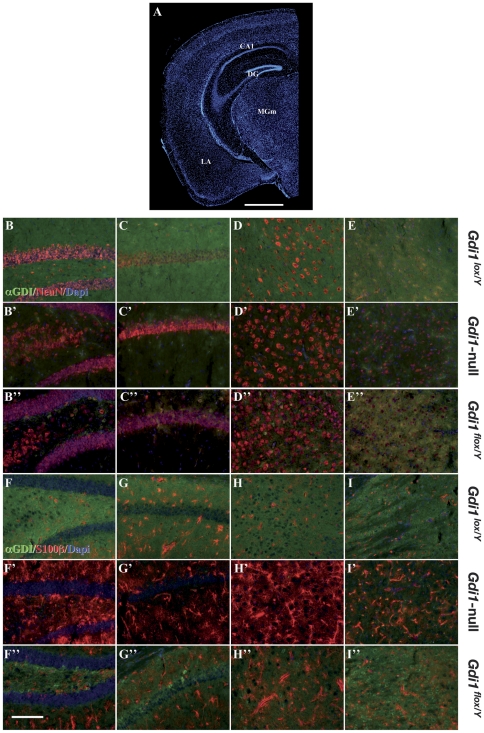
Immunofluorescence analysis of *Gdi1^lox^*, *Gdi1^flox/Y^* and *Gdi1*-null mouse brain regions. Immunofluorescence analysis of 15 µm coronal sections of *Gdi1^lox/Y^*. *Gdi1^flox/Y^* and *Gdi1*-null. (**A**) Low magnification coronal section from *Gdi1^lox/Y^* brain indicating the regions of interest reported on the right: DG (dentate gyrus, **B-B″** and **F-F″**), CA1 (hippocampal CA1 region, **C-C″** and **G-G″**), LA (lateral amygdala, **D-D″** and **H-H″**) and MGm (medial geniculate nucleus, **E-E″** and **I-I″**). (**A**) Scale bar: 1 mm. (**B–I″**) Scale bar: 0.015 mm.

Additionally, double immunofluorescence for αGDI and NeuN on brain slices from *Gdi1^lox/Y^*, *Gdi1^flox/Y^* and *Gdi1*-null mice at PND 56 revealed that αGDI down-regulation in *Gdi1^flox/Y^* was specifically observed in the anterior forebrain, as previously reported [8] (Fig. 3B–E″). Surprisingly, double immunofluorescence for αGDI and glial markers such as S100β (Fig. 3B–E″) or GFAP (data not shown), revealed that αGDI was also down-regulated in S100β and GFAP positive glial cells in *Gdi1^flox/Y^* brain regions.

### *Gdi1^flox/Y^* mice recapitulate the *Gdi1*-null cognitive and behavioral phenotypes

We previously showed that adult *Gdi1*-null mice did not have defects in emotional or exploratory behavior but were selectively impaired in hippocampal-dependent tasks important for acquisition of memory across short time intervals [5]. To assess whether the deletion of αGDI in post-natal life and in forebrain regions plays a key role in associative memory formation, *Gdi1^flox/Y^* and *Gdi1^lox/Y^* male littermates were subjected to a battery of behavioral tests at PND 60 in order to assess exploration, learning and social recognition, as previously preformed for *Gdi1*-null mice [5].

#### Exploration tests

Locomotors activity scores did not show significant genotype-dependent differences in the light-dark box, emergence test and the novelty test (data not shown), as previously observed for *Gdi1*-null mice [5].

### Learning and memory tests

#### Water Maze

*Gdi1^flox/Y^* and *Gdi1^lox/Y^* male littermates behaved normally in the standard hidden-platform version of the water maze. No significant differences between genotypes were observed in all the variables analyzed, confirming that *Gdi1^flox/Y^* mice do not show alterations in spatial memory, as was previously shown for *Gdi1*-null mice [5].

#### Radial Maze

**Figure 4:**
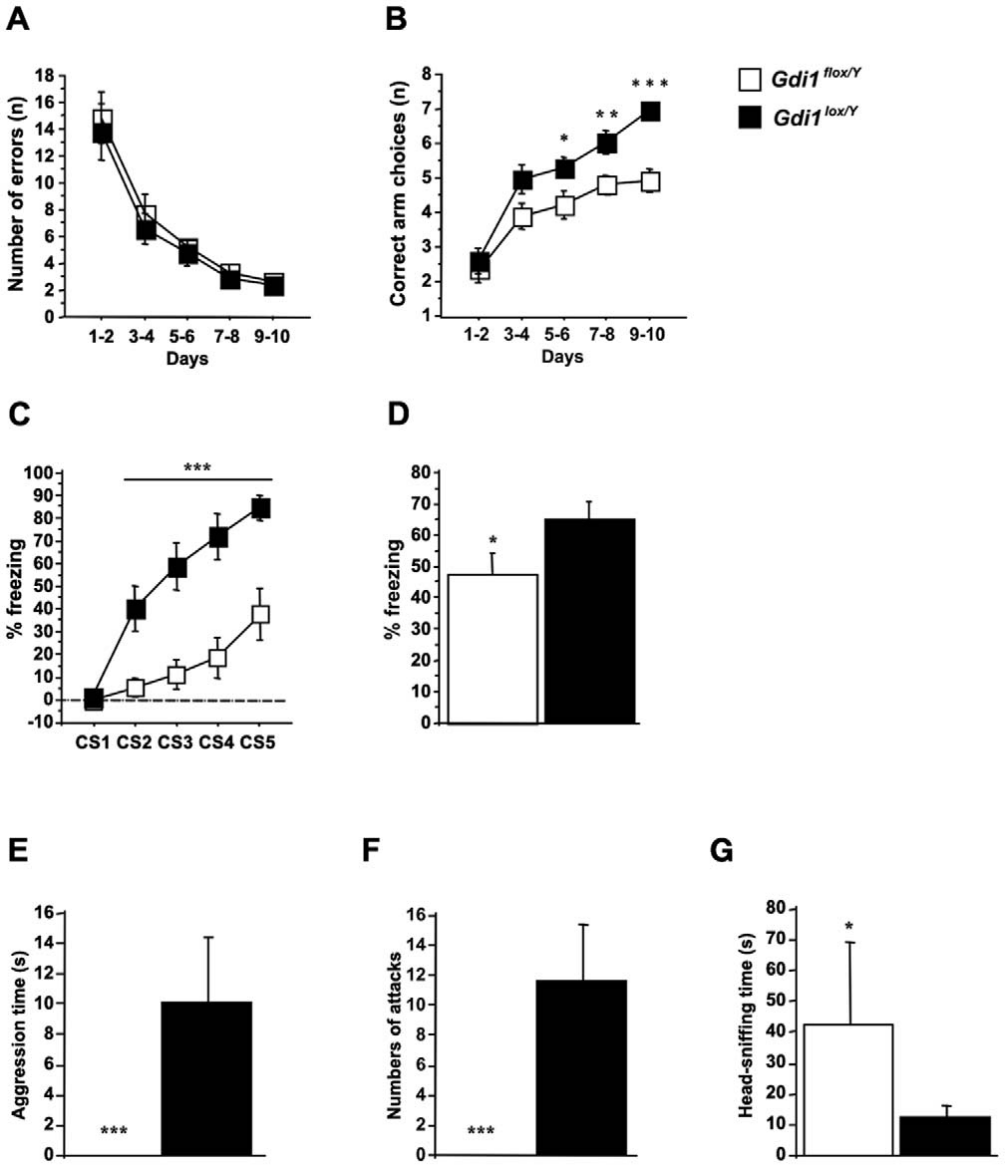
*Gdi1^flox/Y^* mice showed impaired working and associative memory and overfriendly behavior. (**A–B**) *Gdi1^flox/Y^* (n = 14) and *Gdi1^lox/Y^* (n = 12) mice were tested for 10 days in Radial Maze as described in Materials and Methods. (**A**) Mean number of errors until eight correct choices were made. (**B**) Learning performance expressed as the mean number of correct arm choices before the first error. Data points represent the mean ± SE. Black squares are *Gdi1^lox/Y^* animals and white squares are *Gdi1^flox/Y^*. (**C–D**) *Gdi1^flox/Y^* (n = 15) and *Gdi1^lox/Y^* (n = 15) animals were tested in trace fear conditioning. (**C**) Average percentage of freezing displayed during each 15 seconds of CS presentation, during the training session. Data points represent mean freezing ± SE. Black squares are *Gdi1^lox/Y^* animals and white squares are *Gdi1^flox/Y^*. (**D**) Average percentages of freezing during the context memory test. (**E–F–G**) *Gdi1^flox/Y^* (n = 10) and *Gdi1^lox/Y^* (n = 10) mice were tested in the resident intruder test. (**E**) Cumulative duration of the offensive attacks. (**F**) Total number of attacks. (**G**) Cumulative duration of the time spent sniffing the snout area of the intruder. The histograms represent the mean ± SE. *p<0.05, **p<0.01, ***p<0.001.

*Gdi1^flox/Y^* and *Gdi1^lox/Y^* male littermate mice showed altered spatial working memory as assessed by the radial maze test. The number of total errors declined over the ten days of training in both *Gdi1^flox/Y^* and *Gdi1^lox/Y^* mice, and no difference between genotypes was observed (ANOVA genotype by repeated exposure F[1,24] = 0.545; p = 0.467; Fig. 4A). *Gdi1^flox/Y^* and *Gdi1^lox/Y^* mice had significantly different performance in the position of the first repetition (ANOVA F[1,24] = 21.47, p = 0.0001). Although *Gdi1^lox/Y^* mice eventually reached nearly perfect performance (seven out of a maximum number of eight correct successive arm visits), *Gdi1^flox/Y^* mice barely scored above chance level (5.5 correct successive arm visits after 10 days of training) (Fig. 4B). In conclusion, the *Gdi1^flox/Y^* mice seem to have no defects in procedural learning but do have a specific and severe deficit in working memory, as was seen in *Gdi1*-null mice [5].

#### Fear Conditioning

Mice were also tested for contextual and trace fear conditioning. No differences were observed between *Gdi1^flox/Y^* and *Gdi1^lox/Y^* mice in the ability to associate the CS with the US during the training session (ANOVA genotype effect: F[1,40] = 0.208, p = 0.65) as well as 24 h later when testing the context (ANOVA genotype effect: F[1,40] = 0.008, p = 0.929) and cue memory test (ANOVA genotype effect: F[1,40] = 0.239, p = 0.628) in the contextual fear conditioning (data not shown). Instead, as previously shown for the *Gdi1*-null mice [5], there was a significant difference between genotypes in trace fear conditioning when animals were tested for freezing in the training session (ANOVA genotype effect: F[1,24] = 12.5, p = 0.001) and tested for context (ANOVA genotype effect: F[1,24] = 4.8, p = 0.004), as shown in Fig. 4C, D.

#### Resident-intruder test

*Gdi1^flox/Y^* and *Gdi1^lox/Y^* mice were tested in the resident-intruder test. Significant difference between genotypes was observed in the total aggression time (ANOVA genotype effect: F[1,8] = 24.4, p = 0.001), the number of attacks (ANOVA genotype effect: F[1,8] = 38.7, p = 0.0003) and in the time spent sniffing the head of the intruder (ANOVA genotype effect: F[1,8] = 7.1, p = 0.03) (Fig. 4E–G).

In conclusion, all of these data suggest that the down-regulation of αGDI in adult forebrain regions is sufficient to recapitulate the learning deficit previously shown in *Gdi1*-null mice [5]. These results suggest that αGDI acts via RAB GTPase availability to play a pivotal role in *Gdi1*-dependent short-term memory formation and maintenance.

### A defect in excitatory transmission onto lateral amygdala principal cells in the absence of αGDI points to a defect in fast SV recycling

Glutamatergic lateral amygdala (LA) principal cells constitute the vast majority of neuronal cells in this amygdala nucleus [9] and can be easily identified by their electrophysiological properties [10]. They receive not only excitatory synapses from incoming projections from the cortex (cortico-LA synapses) and the medial division of the medial geniculate nucleus of the thalamus (MGm, thalamo-LA synapses) [11] but also input from neighboring principal cell collaterals. To investigate whether the emotional-related brain structures such as the amygdaloid nucleus may have a role in the fear-related behavior observed in the mutant mice, synaptic plasticity was analyzed at those synaptic connections.

**Figure 5:**
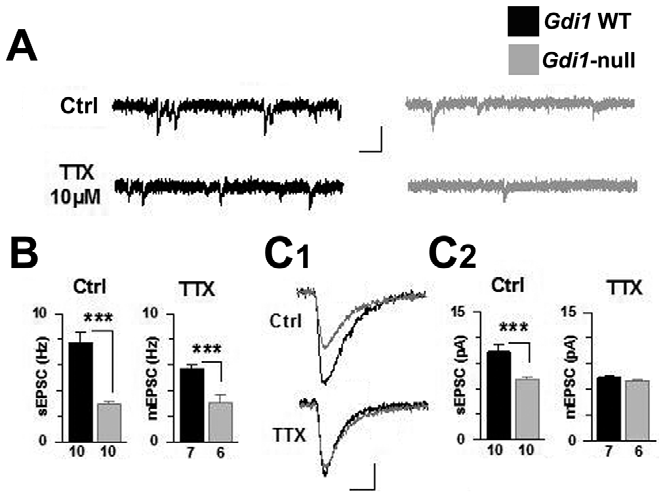
Excitatory transmission onto LA principal cells of *Gdi1*-null mice. (**A**) Spontaneous (Ctrl) and miniature (TTX) EPSCs were recorded in LA principal cells from *Gdi1* WT and *Gdi1*-null littermates. Scale bars: 20 pA and 60 msec. (**B**) Mean EPSC frequency is lower in LA cells from *Gdi1*-null mice in absence or presence of TTX. The number of recorded cells is indicated. ***p<0.001. (**C**) Decrease of spontaneous – but not miniature - EPSC amplitude in absence of αGDI. (**C_1_**) Spontaneous EPSC (Ctrl) and miniature EPSC (TTX) in both genotypes. Scale bars: 5 pA and 10 msec. (**C_2_**) Mean EPSC values for each conditions. The number of recorded cells is indicated.

To better define the previously described hippocampal short-term synaptic defect [6] and to provide a comparison between *Gdi1^flox/Y^* and *Gdi1*-null mice, the overall number of excitatory synaptic contacts impinging onto LA principal cells in juvenile (PND 28) *Gdi1*-null and *Gdi1* WT littermates was first examined by measuring spontaneous and miniature EPSCs (in presence of 10 µM Tetrodotoxin) (Fig. 5A, B). In control conditions, we observed a strong reduction in both the frequency (*Gdi1* WT: 7.75±0.85 Hz; *Gdi1*-null: 2.93±0.26 Hz, p<0.001, n = 10 cells in both conditions) and the amplitude of recorded EPSCs (*Gdi1* WT: 10.33±0.80 pA; *Gdi1*-null: 7.02±0.39 pA, p<0.001, Fig. 5B, C_2_). Interestingly, a significant decrease in EPSC frequency persisted in presence of 10 µM TTX (mEPSCs: *Gdi1* WT: 5.71±0.35 Hz, *Gdi1*-null: 3.08±0.63 Hz, p<0.001, Fig. 5B). In contrast, mean mEPSC amplitude was similar in control and mutant animals (*Gdi1* WT: 7.44±0.11 pA; *Gdi1*-null: 6.96±0.13 pA, p>0.05, Fig. 5C_2_). Taken together, these results indicate that in *Gdi1*-null slices, there is little multi-vesicular release and little of spontaneous discharge activity of incoming excitatory axons.

A decrease in mEPSC frequency can result directly from a decrease in the size of the receptor field of LA principal cells, either due to a diminished dendritic tree or due to a lack of dendritic spines. To address these points, we first applied depolarizing steps to assess the cellular capacitance, but we failed to detect any difference in the cell size between genotypes (Capacitance: *Gdi1* WT: 25.5±3.4 pF; *Gdi1*-null: 19.2±1.95 pF, p>0.05, n = 24 and 35 cells, respectively), suggesting that the main morphological properties were conserved and that probably the receptor field was similar in principal cells from *Gdi1*-null and control mice. Then, to directly visualize dendritic spines, we filled the recorded LA principal cells with 10 µM Alexa-488 while acquiring confocal fluorescence pictures of level III/IV dendrites using 2-photon laser scanning microscopy [12] but again did not observe any differences between *Gdi1* WT and *Gdi1*-null mice (data not shown). These data suggest the presence of a lower release probability and/or a lower number of functional excitatory synapses onto LA principal cells but a normal quantal size in *Gdi1*-null mice.

### Alteration of cortico-LA synaptic weight in the absence of αGDI

**Figure 6:**
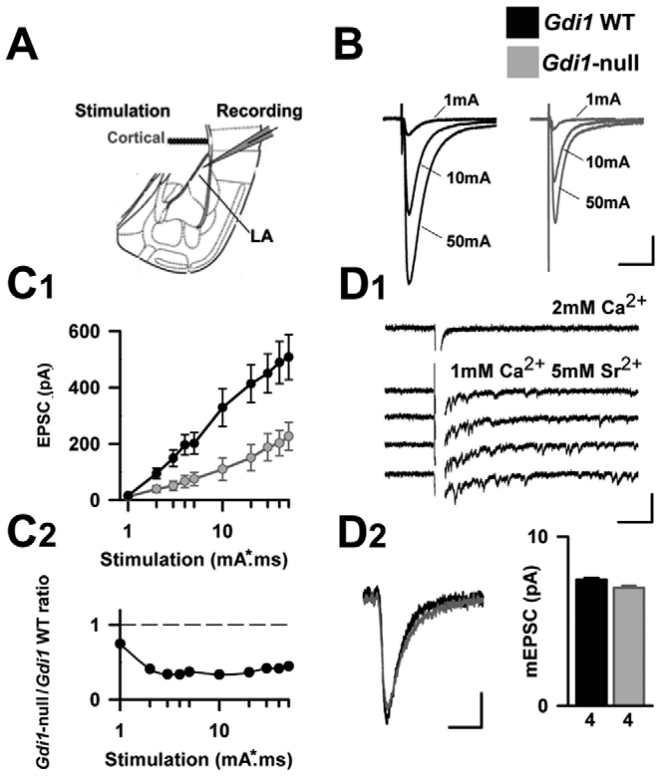
Synaptic transmission at cortico-LA synapses is affected by the absence of αGDI. (**A**) Scheme of the slice preparation. (**B**) Cortico-LA synaptic currents were elicited by stimulations in the external capsule. Traces displayed typical responses for increasing strength (in mA/msec). Scale bars: 100 pA and 15 msec. (**C_1_**) Recorded EPSC amplitude were averaged respective to the genotype and stimulation strength. (**C_2_**) For each stimulation intensity a ∼60% decrease of EPSC size is observed in *Gdi1*-null mice. (**D_1_**) Miniature EPSCs at cortico-LA synapses were obtained by perfusion of Sr^2+^ containing ACSF. Desynchronized EPSCs were analyzed in the tail of evoked EPSCs. Scale bars: 50 pA and 50 msec. (**D_2_**) The average time course (left) and amplitude (right) of desynchronized EPSCs were similar between *Gdi1*-null and *Gdi1* WT mice. Number of recorded cells is indicated. Scale bar: 3 pA and 10 msec.

Cortical projections to the LA can be directly activated by positioning a stimulation electrode within the external capsule in coronal LA-containing acute slices of mouse brain (Fig. 6A) [13]. The application of a short (1 ms) current pulse elicits a monosynaptic EPSC whose amplitude increases progressively with the stimulation intensity (Fig. 6B). At the maximal stimulation intensity - determined as not being noxious for the incoming fibers, evoked EPSC (eEPSC) amplitude reaches a maximal value (eEPSC_max_, Fig. 6B). Interestingly, the input/output (I/O) relationships obtained in *Gdi1* WT and *Gdi1*-null animals were different. Indeed, the first eEPSC was obtained at similar stimulation intensities, indicating that the AP-threshold may be similar in both genotypes, while the amplitude of the eEPSC at supra-threshold intensities was systematically lower in null mice (at 10 mA*ms: *Gdi1* WT: 328±67 pA; *Gdi1*-null: 110±41 pA, p<0.01, n = 8 and 14 cells, respectively, Fig. 6C_1_). Furthermore, when extrapolated, the I/O curves from both genotypes gave different eEPSC_max_ values, suggesting that the synaptic weight of cortico-LA synapses was altered in the absence of αGDI (eEPSC_max_: *Gdi1* WT: 491±74 pA; *Gdi1*-null: 217±46 pA, p<0.01).

In accordance with previous results, the quantal size (*Q*) at cortico-LA synapses was unaffected in the absence of αGDI. Indeed, by replacing part of the external Ca^2+^-concentration with strontium ions (Sr^2+^), a “loose” synchronization of vesicular fusion is usually obtained, allowing the analysis of currents associated with single fusion events in the tail of the main EPSC (Fig. 6D_1_). A careful analysis of these desynchronized events showed that mEPSC amplitude at cortico-LA synapses was comparable in both genotypes (Sr^2+^ mEPSC: *Gdi1* WT: 7.33±0.8 pA; *Gdi1*-null: 7.22±0.48 pA, p>0.05, n = 4 cells in each condition) and resembled global miniature activity (Fig. 6D_2_). Thus, alteration of the I/O curve did not seem to result from a change in quantal size.

### Modification of some pre-synaptic parameters at cortico-LA synapses absent in αGDI

Short-term synaptic plasticity is essentially determined by residual calcium in the synaptic terminal and by the rate at which release sites are refilled by fusion-competent synaptic vesicles [14]. The refilling rate can be assessed by high-frequency iterative stimulations: after the consumption of the pool of release-ready vesicles (RRP), neurotransmitter (NT) release usually stabilizes. The plateau level is then determined by the “refilling rate”, which is mostly dependent on vesicular recycling at the synaptic terminal [15].

**Figure 7:**
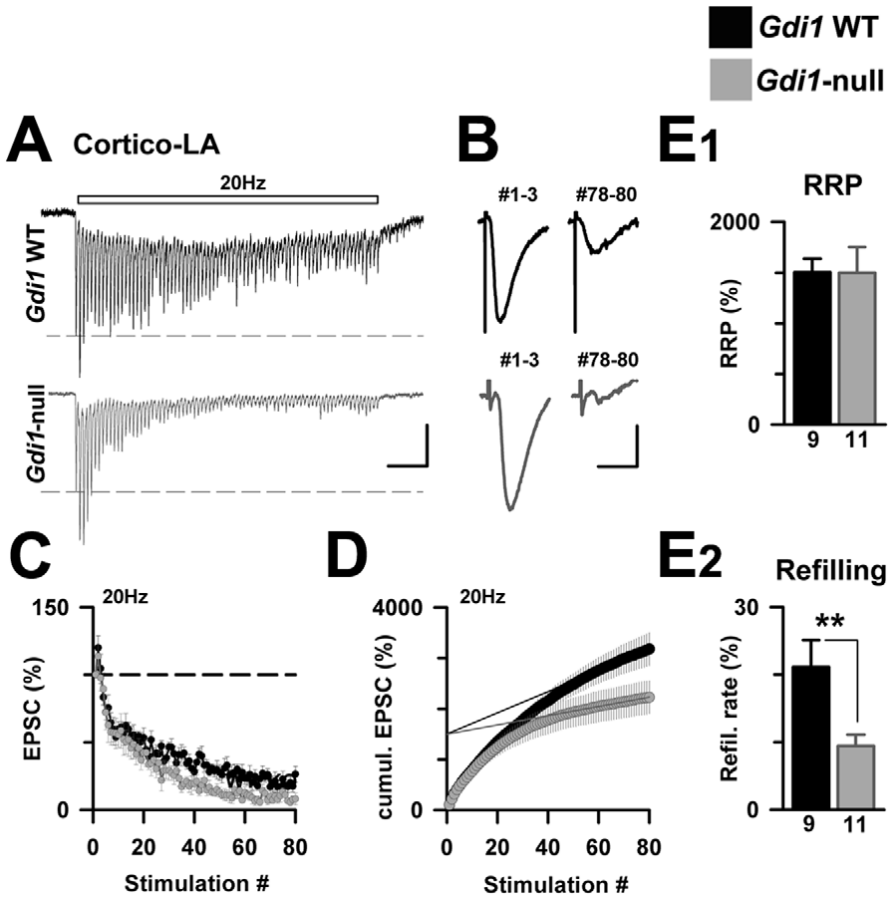
Short-term plasticity at Cortico-LA synapses is perturbed in absence of αGDI. (**A**) Iterative stimulations (20 Hz/4 sec) were applied at Cortico-LA synapses in *Gdi1* WT and *Gdi1*-null mice. Scale bars: 50 (up) and 100 (bottom) pA and 250 msec. (**B**) The pronounced synaptic depression in *Gdi1*-null mice is well illustrated by the extraction of the last EPSCs of the train response. Scale bars: 50 (up) and 100 (bottom) pA and 10 msec. (**C**) Mean EPSC amplitude at a given stimulation for each genotype. (**D**) Cumulative plot based on the same data, allowing to visualize the deficit in refilling rate at *Gdi1*-null synapses (see material and methods section for further details). (**E_1_**) Ready releasable pool size (RRP) and refilling rate (**E_2_**) were calculated in *Gdi1*-null and *Gdi1* WT preparations and expressed as % of initial EPSC size. Number of recorded cells is indicated. **p<0,01.

Thus, we looked at vesicular pool dynamics by applying 20 Hz/4 sec stimulation trains at cortico-LA synapses from *Gdi1* WT and *Gdi1*-null animals. At a typical WT synapse, NT release first facilitates and then progressively decreases to stabilize at ∼20% of the initial EPSC amplitude (Fig. 7B, C) [16]. The absence of αGDI led to major changes in the postsynaptic response to 20 Hz trains; after a first phase of facilitation due to calcium accumulation, the synaptic depression was exacerbated, and the final plateau level was significantly lower compared to *Gdi1* WT littermates (Fig. 7B, C). Both RRP size and refilling rate were extracted from cumulative plots of EPSC amplitude (Fig. 7D, E_1_, E_2_) [15]. In the absence of αGDI, the refilling rate was strongly depressed (*Gdi1* WT: 21.1±4% of initial EPSC; *Gdi1*-null: 9.4±1.7% of the initial EPSC, n = 9 and 11 cells, respectively, p<0.05, Fig. 7E_2_), whereas the RRP size was preserved (*Gdi1* WT: 1503±136% of initial EPSC; *Gdi1*-null: 1498±257% of the initial EPSC, p>0.05, Fig. 7E_1_). Finally, we noticed that no post-tetanic potentiation was present at *Gdi1*-null synapses (data not shown), possibly because of the higher depression observed during high-frequency trains. The lack of potentiation indicated that synaptic plasticity was deeply affected in the absence of αGDI.

### Conditional *Gdi1* mutation indicates the potential pre-synaptic role of αGDI

As reported above, a progressive decrease in αGDI expression is observed in the conditional *Gdi1* mouse after 4 weeks of age (PND 28) in the hippocampus, cerebral cortex and amygdala, whereas it remains elevated in the medial division of the medial geniculate nucleus (MGm, Fig. 3E-E″ and I-I″). Thus, the examination of synaptic physiology at thalamo-LA and cortico-LA synapses in 8–10 week-old *Gdi1^flox/Y^* animals, i.e., a time at which αGDI deletion is effective, directly examines the consequences of pre- and post-synaptic absence of αGDI at excitatory synapses.

**Figure 8:**
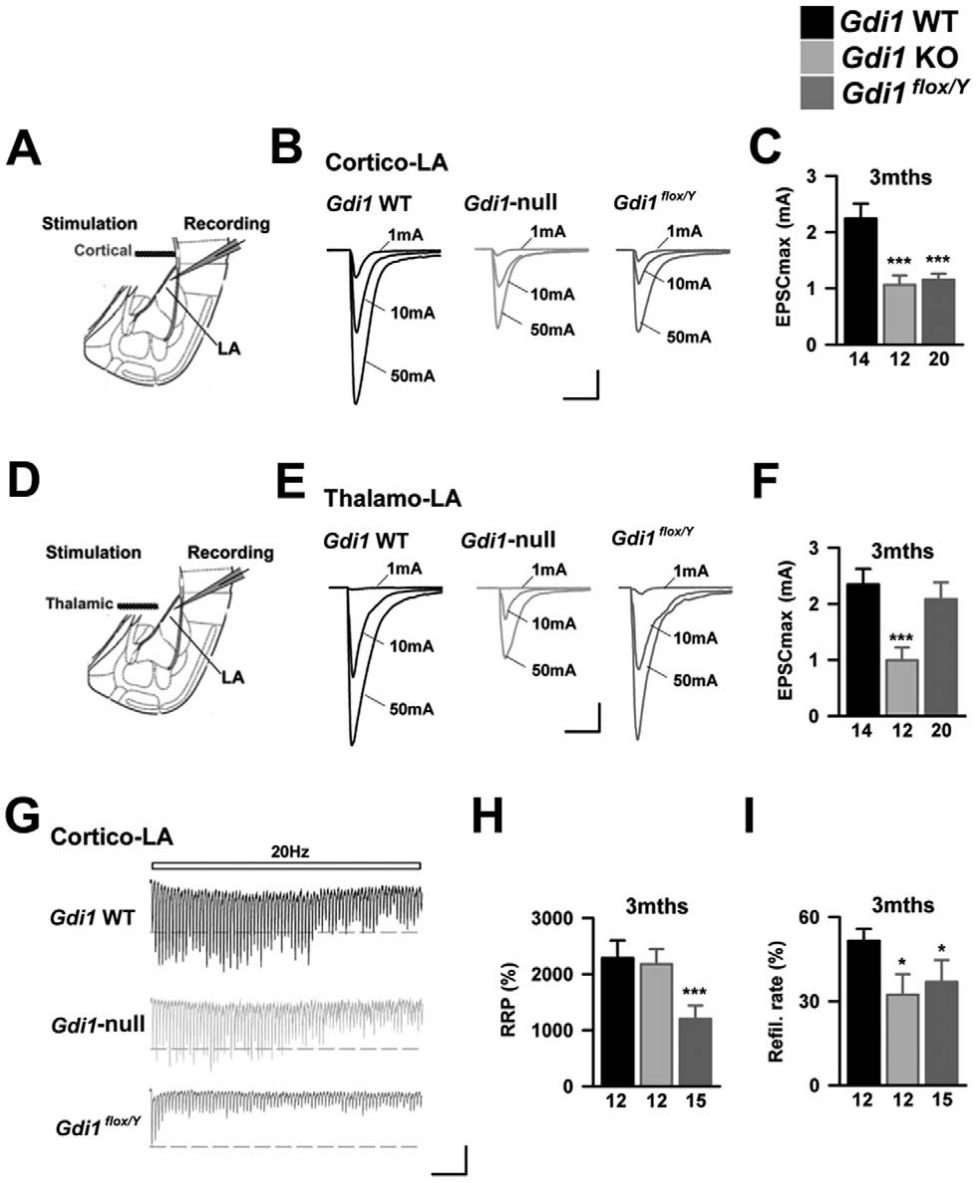
Delayed *Gdi1* deletion leads to exaggerated synaptic phenotypes. (**A**) Scheme of the slice preparation (**B**) Evoked EPSCs obtained in *Gdi1* WT, *Gdi1*-null and *Gdi1^flox/Y^* for different stimulation intensities were recorded. Scale bars: 500 pA and 15 msec. (**C**) Bar graph presenting the mean EPSC amplitude at maximal stimulation intensity (50 mA*msec, defined as EPSCmax) in the various genotypes. Number of recorded cells is indicated. ***p<0.001. (**D–F**) Same presentation as in A–C for the examination of Thalamo-LA synapses. (**G**) Representative postsynaptic responses to high frequency trains of stimulation in 3 months old *Gdi1* WT, *Gdi1*-null and *Gdi1^flox/Y^* animals. Scale bars: 50 pA and 500 msec. (**H**) Calculated RRP size was decreased in *Gdi1^flox/Y^* whereas normal in *Gdi1*-null mice. ***p<0.001 (**I**) The refilling rate was lowered in both null and conditional *Gdi1* mutant mice. *p<0.05.

In PND 100 animals, we first compared I/O curves at the two major excitatory entries to the LA by apposing stimulation electrodes in the external and internal capsules (Fig. 8A, D) [13]. In *Gdi1* WT animals, the I/O curves reach an eEPSC_max_ of ∼2 mA at both stimulated pathways (cortico-LA: 2238±272 pA, thalamo-LA: 2345±279 pA, Fig. 8C, F). Similar to previous results in P100 *Gdi1*-null animals, both I/O curves were strongly affected (eEPSC_max_: cortico-LA: 1063±164 pA, thalamo-LA: 995±224 pA, both p<0.001 as compared to *Gdi1* WT). A normal I/O relationship was observed in P100 *Gdi1^flox/Y^* mice in the thalamo-LA pathway (*Gdi1^flox/Y^*: 2083±298 pA, p>0.05 as compared to *Gdi1* WT, Fig. 8 F), indicating that postsynaptic αGDI was not crucial in determining synaptic efficacy at these synapses and that the deficit observed in the constitutive null mice was due to the pre-synaptic absence of αGDI. In contrast, the delayed absence of αGDI at cortico-LA synapses was associated with a strong alteration in the I/O curve (*Gdi1^flox/Y^*: 1155±103 pA, p<0.001 compared to *Gdi1* WT, Fig. 8C), which mimicked the constitutive mutation. Thus, these results show that the presence of pre-synaptic αGDI is essential in maintaining normal excitatory projections to the LA.

### Delayed absence of αGDI causes additional pre-synaptic deficits

We next compared short-term plasticity at cortico-LA synapses in PND 100 *Gdi1* WT, *Gdi1-null* and *Gdi1^flox/Y^* mice using trains of 80 stimulations 20 Hz in magnitude (Fig. 8G). Post-synaptic responses to high frequency trains were affected at null synapses and were characterized by higher fatigue compared to WT synapses (Fig. 8G). However, analysis of cumulative EPSCs showed a major difference between both mutant mice: as seen in young animals (Fig. 7), a decrease in the refilling rate is present in not only PND 100 *Gdi1*-null mice but also *Gdi1^flox/Y^* PND 100 mice (Fig. 8I). More surprisingly, the RRP size of *Gdi1^flox/Y^* cortico-LA synapses was found to be significantly lower than in *Gdi1* WT and *Gdi1*-null mice (*Gdi1* WT: 2287±312% of initial EPSC, *Gdi1*-null: 2181±270%, *Gdi1^flox/Y^*: 1201±239%, p>0.05 for both, Fig. 8H). This unexpected observation may indicate the existence of cellular mechanisms that correct the synaptic dysfunction when the mutation occurs during early development.

## Discussion

We previously showed that *Gdi1*-null mice had a specific hippocampus-dependent short-term memory impairment and overfriendly behavior [5]. We suggested that the memory deficit might be caused by alterations in the SV pool and short-term synaptic plasticity involving alterations of different trafficking pathways that are controlled by several RAB GTPase proteins [6].

A major limitation for the study of αGDI function in the central nervous system (CNS) is that *Gdi1* is ubiquitously expressed and that complete ablation of *Gdi1* occurs in all brain regions and all brain cell types throughout development in the null mice.

In this study, we used the CRE/lox *P* recombination system to spatially and temporally restrict knockout of the *Gdi1* gene to postmitotic neurons and glia of the anterior forebrain.

Our results show that *Gdi1^flox/Y^* mice, in which *Gdi1* is strongly reduced from PND 56 in the hippocampus, cortex and amygdala, were viable and fertile, and unlike the *Gdi1*-null mice, they did not present visible morphological or neuro-pathological phenotypes. The learning and memory and behavioral characterization of these mice showed no difference in emotion, explorative behavior, spatial memory or contextual fear conditioning. In contrast, *Gdi1^flox/Y^* mice suffered from a specific short-term memory deficit, as indicated by alterations in working memory in the radial maze test and associative fear-related memory assessed by trace fear conditioning. Moreover, we also observed loss of aggressive behavior, as previously demonstrated for the *Gdi1*-null mutants [5]. Taken together, these data suggest that postnatal inactivation of *Gdi1* specifically in these brain regions is sufficient and recapitulates the cognitive and social deficits previously seen in *Gdi1*-null mice.

We then investigated the modifications in synaptic plasticity at different synaptic connections in the cortico-lateral amygdala (LA) of P30 *Gdi1*-null mice. Previous studies have reported that cortical projections to the LA contribute to emotional learning such as fear-related memory, providing qualitative information in addition to the highly responsive sub-cortical pathway [9]. Thus, we examined cortico-LA synaptic physiology in the absence of αGDI by evaluating synaptic depression in the cortico-amygdala pathway. Our results show a strong reduction in the overall synaptic weight at these synaptic contacts, associated with alterations of pre- but not post-synaptic parameters, suggesting that there are specific alterations in the refilling rate of release sites.

The examination of synaptic physiology at thalamo-LA and cortico-LA synapses in adult *Gdi1^flox/Y^* animals provides the opportunity to directly examine the consequences of pre- and post-synaptic absence of αGDI at excitatory synapses. The first important finding was that in contrast to *Gdi1*-null mutants, the maximal thalamo-LA EPSC was not changed in conditional *Gdi1^flox/Y^* mice (Fig. 8). Taken together with the results showing that the number of active cortico-LA synapses is lowered both in conditional and *Gdi1*-null mice, this suggests that presynaptic but not post-synaptic *Gdi1* is important in maintaining the integrity of excitatory projections to the LA. Given the strong link between the physiology of excitatory projections to the LA and emotional learning, it is likely that these synaptic defects participate in the fear-related learning deficit that is seen in αGDI mutant mice. In addition to the functional disappearance of excitatory synapses, we also observed that the ready releasable pool size at the remaining cortico-LA synapses was significantly decreased in conditional but not *Gdi1*-null mutants (Fig. 8). This possibly suggests that if αGDI deletion occurs during the early phases of synaptogenesis, its absence may be overcame molecularly or functionally, which would no longer be possible in the adult consolidated synapses. Physiological experiments conducted on adult cortico-LA synapses suggest some functional differences. For example, we observed that the release probability at cortico-LA synapses, which is modified by classical fear conditioning procedures [17], is significantly higher in adult animals. Interestingly, this was observed together with a reduction in pre-synaptic expression of long-term plasticity (Y. Humeau F. Gambino, personal communication). Taken together, these observations suggest that during the life of the animal, experience-driven consolidation and maturation at excitatory synapses occur in the lateral amygdala, a phenomenon that depends on the presence of αGDI and possibly specific RAB GTPases involved in SV exo- and endocytosis.

We previously suggested that the lack of αGDI seems to perturb the functionality of different SV pools at the pre-synaptic site and may be due to alterations of at least two different classes of RAB GTPases, the SV-associated RAB3A and the endosomal RAB proteins RAB4 and RAB5 by altering cycling between the active GTP- and inactive GDP-bound state. Despite knowledge about the mechanisms by which RABs regulate intracellular exo- and endocitic events, it is not clear how many RABs are involved in these pathways and whether all of them are susceptible to αGDI-mediated regulation. Recently, Pavlos et al. [7], use d a combination of different techniques to conclude that SVs contained a set of RABs associated with SV membranes involved both in exocytosis and endosomal recycling, including RAB3A/B/C, RAB4B, RAB5A, RAB10, RAB11B, RAB14 and RAB27B. They also showed that RAB27B exhibits both overlapping and distinct features from RAB3.

In conclusion, our results point to a defect in pre-synaptic function that affects several parameters of short-term synaptic plasticity involved in hippocampus-related short-term memory deficits as well as in amygdala-related behavior. Stronger defects in short-term synaptic plasticity are present when *Gdi1* is deleted specifically in the forebrain during adult life, suggesting a predominant role of *Gdi1* via a subset of specific RAB GTPases acting specifically in these brain regions at the pre-synaptic sites and leading to learning and social alterations. In this regard, our results encourage further studies to identify RAB cell specificity and function in neurons and glial cells, which will most likely shed light on the RABs that determine the *Gdi1* learning phenotype.

## Materials and Methods

### Animals

All animals were maintained on a 12 hrs light/darkness cycle at 22°–25°C. Food pellets and water were available ab libitum.

Experiments were done according to the animal protocols approved by the Institutional Animal Care and Use Committee San Raffaele (IACUC) (San Raffaele, Milan, Italy) and were approved by the National Ministry of Health, IACUC ID 470. All experiments were carried out in accordance with the guidelines established by the European Community Council Directive of 24 November 1986 on the use of animals in research (86/609/EEC). All efforts were made to minimize animal suffering and to use only the number of animals necessary to produce reliable results.

### Behavioural tests

#### Dark/light box

A 20×30-cm lit chamber with transparent Perspex walls (20 cm high) and open top was connected to a 20×15×20-cm plastic dark box which was completely closed except for the 7.5×7.5-cm door connecting it to the lit chamber. Illumination was by direct room light (500 lx). Each mouse was released in the middle of the lit compartment and observed for 5 min.

#### Novel object test

Frames of non-reflective aluminium (37 cm high) were used to partition a round open field arena (diameter of 150 cm and 35-cm high walls) into four squares 50×50-cm arenas, allowing for concurrent observation of four animals. Illumination in the room was by indirect diffuse room light (4×40-W bulbs, 12 lx). The novel object was a 50-mL Falcon tube positioned vertically in the center of the arena. Each animal was observed for 30 min in the empty arena as pre-exposure. The novel object was then introduced and observation continued for another 30 min.

#### Emergence test

It is used the same arena used in the Novel object test. In the mouse cage the day before a box made of plastic (12×8×4 cm with opening of 8×4 cm) is inserted as home and during the test it is placed in a corner of the arena, at 5 cm from the nearest walls, with the opening facing away from the wall. The mice were observed for 30 min. For time course analysis, the total observation time was portioned into six periods of 10 min.

#### Water maze

The standard hidden-platform version of the water maze was done as previously described [5]. Briefly, the test included an acquisition phase (18 trials, six/day, inter-trial time 30–40 min) followed by a reversal phase during which the platform was moved to the opposite position (12 trials, six/day). For the analysis the trials were averaged in blocks of two trials.

#### Radial Maze

The apparatus consisted of eight arms (38 cm long, 7 cm wide) extending from an octagonal centre platform (diameter 18.5 cm) with 5-cm transparent plastic walls. The distance from the platform centre to the end of each arm was 47 cm. At the end of each arm is present a cup with a food pellet. Food-deprived mice (maintained at 85% of their free-feeding weight) were placed in the center platform and allowed to collect pellets placed at the end of each arm for 10 min. The animals were adapted to the maze for 1 day and then tested for 10 days. For each trial, the total number of arm choices, number of correct choices before the first error, total number of errors was recorded.

#### Fear conditioning test

Auditory trace fear conditioning were performed as previously described [5]. All mice were pre-exposed to the test chamber (Ugo Basile, Italy) for 10 minutes on the two days preceding conditioning. During the training session, trial started with the presentation of the CS (15 s), followed 15 s later by the presentation of the shock for 2 s. This was repeated 5 times with a 60 seconds inter-trial intervals (ITI).

Twenty-four hours after fear conditioning, mice were placed in the conditioning box again, measuring their freezing behavior in the context test (2 min without CS “contextual freezing”). During the test, animals were video-tracked using the ANY-maze system (Anymaze, Stoelting Co, Wood Dale, IL, USA, www.anymaze.com).

#### Resident-intruder test

Resident adult male mice were kept individually for 2 weeks prior to the test and were used as residents. C57BL/6N mice, housed in groups of 5, were used as intruders. After transfer of the intruder to the home cage of the resident the behavior was tape-recorded and the cumulative duration of aggressive behavior (biting attacks, tail rattling) and social behavior (sniffing head or back, social grooming) were determined.

### Video tracking, data collection and statistical analysis

During all the tests, animals were video-tracked using the EthoVision 2.3 system (Noldus Information Technology, Wageningen, the Netherlands, http://www.noldus.com) using an image frequency of 4.2/s. Raw data were transferred to Wintrack 2.4 (http://www.dpwolfer.ch/wintrack) for off-line analysis. Statistical computations were done using Statview 5.0 (SAS Institute, Cary, NC, USA).

### Gene targeting

The entire mouse sequence of *Gdi1* was isolated from the 129/SvEv mouse genomic library and sequenced (accession number AF441240), as previously described [5]. The targeting vector was constructed as follow: the 5′ arm consists on a KpnI/NdeI 1625 bp fragment containing the 1^st^ exon and part of the 1^st^ intron of *Gdi1* gene; lox *P* sites are inserted in the 1^st^ and in the 3^rd^ intron, in order to delete the *Gdi1* gene between exon 2 and 3; the neomycin-resistance (neo) cassette, driven by promoter and 5′ UTR sequence from the mouse *Pgk1* gene, flanked by *frt* sites was inserted in opposite orientation in the 3^rd^ intron, immediately downstream of the 3′ lox *P* site; the 3′ arm of the targeting vector consists on EcoRV/EcoRI 7 kb containing the last 8 exons of *Gdi1* and part of the 5′ region of the flanking gene.

After linearization with unique NotI restriction site and electroporation in AB1 embryonic stem (ES) cells, G418-resistant colonies were analyzed by PCR, from primers F4 and NEO1C with XL Expand Boheringher.

PCR positive clones were confirmed by Southern blot. The 5′ flanking probe S1 was an RT-PCR product of 320 bp containing exons 6 and 7 of the 16A 5′ flanking gene. A 590 bp probe containing the genomic region cloned between lox *P* sites was used to confirm the insertion of both sites. A neo probe, obtained by PCR from primers neo2 and neo6, was used to confirm the insertion of neo cassette. One of three homologous recombinant clones was injected into blastocysts by standard methods and chimera mice were generated. Chimera female mice were crossed to C57Bl/6N mice. Genotypes were determined by PCR from tail DNA biopsy using primers Lox1 and Lox2. The primer sequence is showed in table S1.

### Generation of *Gdi1^flox^* mice

Heterozygote *Gdi1^lox/X^* female mice were crossed with transgenic SJL-TgN(ACTFLPe)9205Dym mice (Jackson Laboratory, Bar Harbor, ME, USA) expressing a variant of the *S. cerevisiae* FLP1 recombinase gene under the human b-actin promoter to delete the neo cassette. Mice were analyzed by PCR analysis of DNA extracted from the tail using primers Neo4 and Neo5, FlpF and FlpR. Heterozygote *Gdi1^lox/X^* female mice, *neo^−^*, were crossed with CaMKII-CRE-159 transgenic mouse [8], expressing the CRE recombinase under the control of the mouse αCaMKII promoter. Mice genotype was analyzed by PCR analysis of DNA extracted from the tail using primers Lox1 and Lox2, and cre1 and cre2. The primer sequence is showed in table S1.

### Western blot analysis

Brain regions were dissected from *Gdi1^flox/Y^* (n = 3) and *Gdi1^lox/Y^* (n = 3) littermates. Proteins in sample buffer were fractionated in 10% SDS-PAGE, and Western blots were done using standard methods.

### Immunofluorescence analysis

The animals were anesthetized with 2-2-2 Tribromoethanol (20 ml/gr) and transcardially perfused with 20 ml eparin in PBS1X (30 mg/100 ml), and brains were fixed in 4% (w/v) paraformaldehyde (Fluka, Buchs, Switzerland).

Brains were post-fixed in the same fixative overnight at 4°C. Frozen coronal sections (15 µm thick) were established for standard immunofluorescence. Briefly, sections were washed three times with PBS 1X and then incubated in blocking buffer (PBS1X, FBS 10%, BSA 1 mg/ml and Triton 0.1%). Primary antibodies were diluted in blocking buffer and applied on sections at at the following concentration: αGDI (1∶100, Zymed), NeuN (1∶1000, Chemicon), S100β (1∶100, SIGMA), GFAP (1∶1000, Dako), Primary antibodies were incubated over night at +4°C. Section were then washed three times in PBS 1X and secondary antibodies were applied accordingly manufacturer, instructions, nuclei were stained with Dapi. Images were acquired directly from slices using the Olimpus BX51 microscope and 20× objective.

### Electrophysiology

#### Slice preparation

Standard procedures were used to prepare 330 µm thick coronal slices from *Gdi1*-null and *Gdi1* WT, *Gdi1^lox/Y^* and *Gdi1^flox/Y^* male mouse brains following a protocol approved by the European and French guidelines on animal experimentation. Briefly, the brain was dissected in ice-cold artificial cerebrospinal fluid (ACSF), mounted on an agar block and sliced with a vibratome (Leica VT1200s; Germany) at 4°C. Slices were maintained for 45 min at 35°C in an interface chamber containing ACSF equilibrated with 95% O_2_/5% CO_2_ and containing (in mM): 124 NaCl, 2.7 KCl, 2 CaCl_2_, 1.3 MgCl_2_, 26 NaHCO_3_, 0.4 NaH_2_PO_4_, 18 glucose, 4 ascorbate, and then for at least 45 min at room temperature before being transferred to a superfusing recording chamber. The same procedure was followed for manual brain area microdissections, the samples being immediately frozen in liquid nitrogen.

#### Recordings

Whole-cell recordings from LA principal neurons were performed at 30–32°C in a superfusing chamber as previously described [18]. Neurons were visually identified with infrared videomicroscopy using an upright microscope equipped with a 60× objective. Patch electrodes (3–5 MΩ) were pulled from borosilicate glass tubing and filled with a low-chloride solution containing (in mM): 140 Cs-methylsulfonate, 5 QX314-Cl, 10 HEPES, 10 phosphocreatine, 4 Mg-ATP, and 0.3 Na-GTP (pH adjusted to 7.25 with CsOH, 295 mOsm). For current-clamp experiments, Cs-methylsulfonate was replaced with equimolar K-gluconate. All experiments were performed in the presence of picrotoxin (100 µM). Monosynaptic EPSCs or EPSPs exhibiting constant 10–90% rise times and latencies were elicited by stimulation of afferent fibers with a bipolar twisted platinum/10% iridium wire (25 µm diameter). In all experiments, stimulation intensity was adjusted to obtain baseline EPSC amplitudes of ∼100.

#### Data acquisition and analysis

Data were recorded with a Multiclamp700B (Molecular Devices, USA), filtered at 2 kHz and digitized at 10 kHz. Data were acquired and analyzed with pClamp10.2 (Molecular Devices). In all experiments, series resistance was monitored throughout the experiment, and if it changed by more than 15%, the data were not included in the analysis. Spontaneous and miniature events were collected using a template-based detection run onto a 3–5 minute time period. For amplitude calculation, each event was then fitted using a bi-exponential equation leading to better estimation of event characteristics. For RRP and refilling rate measurements, the last 20 (on 80 in total) EPSC amplitude were used to generate the linear fit [15]. All values are given as means ± standard error of the mean (SEM). Mean values were compared between genotypes using either unpaired Student's *t*-test or Mann-Whitney (MW) test as appropriate.

## Supporting Information

Table S1 **Oligonucleotides name and sequence used in this study.**
(DOCX)
