# Supplementary material for: Forebrain Deletion of αGDI in Adult Mice Worsens the Pre-Synaptic Deficit at Cortico-Lateral Amygdala Synaptic Connections
Source: PLoS One. 2012 Jan 23;7(1):e29763. doi: 10.1371/journal.pone.0029763 (PMC3264564; doi:10.1371/journal.pone.0029763)
Supplement: Table S1 — Oligonucleotides name and sequence used in this study. (DOCX) [file pone.0029763.s001.docx]

**Table S1. Primer’s sequences used in the study.**

F4 5’-GGATCAGGAGACAACCGACAGACGC-3’

NEO1C 5’-CACTTCATTCTCAGTATTGTTTTGCC-3’

neo2 5’-GCAGACTTACAGGACGGATCGATC-3’

neo6 5’-GCCGAGAAAGTATCCATCATGGCTG-3’

Lox1 5’-GGAAGACTTGGAAGCTGAAAGCTTT-3’

Lox2 5’-CATGATGCCAGACAGGATGCATTC-3’

Neo4 5’-GCAATCCATCTTGTTCAATGGCCG-3’

Neo5 5’-AAAGCGCACGTCTGCCGCGCTG-3’

FlpF 5’-CATCAATTGTGGAAGATTCAGCG-3’

FlpR 5’-GAGTATACGTTGTCCTGGCCAC-3’

Cre1 5’-GCCGCATTACCGGTCGATGCAACGA-3’

Cre2 5’-CTGGCAGATGGCGCGGCAACACCATT-3'
